# Pregnancy-Associated Spontaneous Coronary Acute Dissection as a Cause of Sudden Cardiac Death—Autopsy Findings and Literature Review: Is COVID-19 Related?

**DOI:** 10.3390/medicina59071257

**Published:** 2023-07-07

**Authors:** Timur Hogea, Bogdan Andrei Suciu, Laura Chinezu, Klara Brinzaniuc, Emil Marian Arbănași, Ancuța Ungureanu, Réka Kaller, Cosmin Carașca, Eliza Mihaela Arbănași, Vlad Vunvulea, Ioana Hălmaciu, Adrian Vasile Mureșan, Eliza Russu, Claudiu Constantin Ciucanu, Casandra Maria Radu, Carmen Corina Radu

**Affiliations:** 1 Department of Forensic Medicine, George Emil Palade University of Medicine, Pharmacy, Science, and Technology of Targu Mures, 540139 Targu Mures, Romania; timur.hogea@umfst.ro (T.H.); cosmin.carasca@umfst.ro (C.C.); carmen.radu@umfst.ro (C.C.R.); 2 Institute of Forensic Medicine, 540141 Targu Mures, Romania; ioana.halmaciu@umfst.ro; 3 Doctoral School of Medicine and Pharmacy, George Emil Palade University of Medicine, Pharmacy, Sciences and Technology of Targu Mures, 540142 Targu Mures, Romania; emilarbanasi1@gmail.ro (E.M.A.); reka.kaller@umfst.ro (R.K.); 4 Department of Anatomy, George Emil Palade University of Medicine, Pharmacy, Science, and Technology of Targu Mures, 540139 Targu Mures, Romania; bodan.suciu@umfst.ro (B.A.S.); vlad.vunvulea@umfst.ro (V.V.); 5 Department of Histology, George Emil Palade University of Medicine, Pharmacy, Science, and Technology of Targu Mures, 540139 Targu Mures, Romania; 6 Clinic of Vascular Surgery, Mures County Emergency Hospital, 540136 Targu Mures, Romania; adrian.muresan@umfst.ro (A.V.M.); eliza.russu@umfst.ro (E.R.); claudio.ciucanu@gmail.com (C.C.C.); 7 Department of Vascular Surgery, George Emil Palade University of Medicine, Pharmacy, Science, and Technology of Targu Mures, 540139 Targu Mures, Romania; 8 Epidemiology Clinic, Mures County Hospital, 540072 Targu Mures, Romania; hanca.ancuta@gmail.com; 9 Faculty of Pharmacy, George Emil Palade University of Medicine, Pharmacy, Science, and Technology of Targu Mures, 540139 Targu Mures, Romania; arbanasi.eliza@gmail.com; 10 Department of Radiology, Mures County Emergency Hospital, 540136 Targu Mures, Romania; 11 Doctoral School of Biological and Biomedical Sciences, University of Oradea, 1 University Street, 410087 Oradea, Romania; rcasandra1996@gmail.com

**Keywords:** sudden cardiac death, spontaneous acute coronary dissection, pregnancy, autopsy, COVID-19, acute myocardial infarction, histopathology

## Abstract

Sudden cardiac death (SCD) is the leading cause of mortality globally (violent or non-violent), with few to no feasible remedies. The etiopathogenesis of SCD involves a complex and multilayered substrate in which dynamic factors interact with a preexistent cardiovascular pathology, which is often undiagnosed and untreated, leading to the rapid development of cardiac rhythm disorders and cardiac arrest. Cardiovascular disease is a rare but emerging factor in maternal mortality that can be justified by an upward trend in the mean age of pregnant individuals. Spontaneous coronary artery dissection (SCAD) is defined as a non-traumatic and non-iatrogenic separation of the coronary arterial wall by intramural hemorrhage with or without an intimal tear. The resulting intramural hematoma compresses the coronary arteries, reducing blood flow and causing myocardial ischemia. SCAD continues to be misdiagnosed, underdiagnosed, and managed as an atherosclerotic acute coronary syndrome, which may harm patients with SCAD. The latest research shows that individuals who have or have had coronavirus disease 2019 (COVID-19) may also present coagulation abnormalities, so infection with COVID-19 during pregnancy can increase this hypercoagulable condition, thus increasing the risk of SCAD and SCD. This present study reports two cases over 35 years, one being infected with SARS-COV2 one month before the event and the other being tested positive during admission, both asymptomatic, who were declared healthy on periodic clinical evaluations, with pregnancies over 35 weeks, with normal fetal development, which suddenly caused chest pain, dyspnea, and loss of consciousness, required emergency c-sections, and died suddenly after they were performed. In both cases, the cause of death was SCAD on the anterior-descending artery. In both cases, emergency percutaneous coronary intervention was performed. The second part of the study represents a literature overview of SCAD during COVID-19. In addition to pregnancy hormonal changes, other potential hormone-mediated SCAD triggers are still under discussion.

## 1. Introduction

Sudden cardiac death (SCD) represents a non-violent death in the majority of cases with a known or unknown cardiovascular etiology. SCD is defined as death from a cardiovascular disease that occurs within one hour of the onset of symptoms, confirmed by witnesses present, or in less than 24 h without witnesses [1]. It is signaled by an abrupt loss of consciousness with or without prior symptoms such as chest pain, which travels from left arm to neck, shortness of breath, sweating, nausea, vomiting, abnormal heart beating, anxiety, fatigue, and weakness, with death usually occurring under one hour from the onset of these symptoms. When an apparently healthy and active individual dies in a public place or at home with or without witnesses, suspicions arise in the justice system that it was a violent death. The term SCD reflects a sudden stop of cardiac activity followed in a couple of minutes by cerebral death [2]. According to the WHO, it is still regarded as the number one cause of death worldwide (violent or non-violent), with few to no viable solutions available [3]. The etiopathogenesis of SCD has a complex and multilayered substrate in which dynamic factors interact with a preexistent cardiovascular pathology, in most cases undiagnosed and untreated, with the rapid development of cardiac rhythm disorders (most often ventricular tachycardia that evolves into ventricular fibrillation) and cardiac arrest.

Cardiovascular disease is an uncommon but rising cause of maternal mortality in both developing and developed countries, which can be explained by an increase in the average age of pregnant women. Despite the difficult screening protocol, observational studies have indicated that mother’s older age at the time of pregnancy is related with an increase in maternal morbidity and death. Still, systematic research for cardiac disease is usually not performed; at an advanced maternal age, investigations focus on prenatal genetic screening and fetal development. As a result, alongside well-established causes of maternal death (hypertension, obstetrical hemorrhage, and sepsis), undiagnosed cardio-coronary pathologies turn into SCD cases when the autopsy report reveals them.

Spontaneous coronary artery dissection (SCAD) is defined as a non-traumatic and non-iatrogenic separation of the coronary arterial wall by intramural hemorrhage with or without an intimal tear. The resulting intramural hematoma compresses the coronary arteries reducing blood flow and producing myocardial ischemia [4]. SCAD in women of childbearing age is very rare, and acute myocardial infarction (MI) due to coronary atherosclerosis or acute coronary dissection is uncommon [5,6]. The physiological modifications that appear in pregnancy, however, have been shown to increase the risk of an acute MI 3-fold compared with the risk in non-pregnant women of similar age. SCAD is a nonatherosclerotic cause of an acute coronary event that most frequently happens in the prepartum or postpartum period and can lead to SCD [7,8], with the prepartum period also being a life-threatening condition for the fetus. The etiopathogenesis of SCAD describes the formation of a hematoma in the coronary wall, producing obstruction or an intimal-medial wall tear [9]. Studies have shown that coronary wall hematoma is a rare consequence of the changes that appear in the fibrinolytic and coagulation systems that describe the hypercoagulable state of pregnancy and the postpartum period [10]. The latest research presumes that individuals who have or have had coronavirus disease 2019 (COVID-19) may also present coagulation abnormalities, so infection with COVID-19 during pregnancy can increase this hypercoagulable state [11,12], thus increasing the risk of SCAD and SCD.

## 2. Materials and Methods

We report two cases over 35 years old, one being infected with SARS-COV2 one month before the event and the other being tested positive during admission, both asymptomatic, who were declared healthy on periodic clinical evaluations, with pregnancies over 35 weeks and normal fetal development, who suddenly complained of chest pain, dyspnea, and loss of consciousness. They both required emergency coronary stenting and c-sections, and died suddenly after it was performed.

### 2.1. Case 1

A 35-year-old female in apparently good health, pregnant in 34 + 3 weeks with normal fetus development (3 previous pregnancies without problems), without any pathological background or history of alcohol consumption, and a smoker, who checked all her mandatory antenatal visits as normal, was brought to an emergency hospital due to her having sudden chest pain, dizziness, weakness, shortness of breath, and cold sweats, with the diagnosis: “IV pregnancy in 34 + 3 weeks. Viable fetus in cephalic presentation. Intact fetal membranes. Eutocic pelvis. Signs of impending premature labor. Dyspepsia. Hypothyroidism. Tobacco use and addiction”.

At the time of admission, laboratory tests revealed SARS-COV2 virus infection through real-time PCR, leucocytosis with neutrophilia, urinary tract infection (nitrite positive), and pregnancy-induced anemia (Hgb 10.6 g/dL). The fetal ultrasonography values were within normal limits. She immediately began tocolytic, hormonal, and antispastic treatment alongside the prevention of hyaline membrane disease. 

On the second day of hospitalization, the infectious disease consultation stated: “SARS COV2 infection, asymptomatic form. Urinary tract infection” and recommended antibiotic treatment with 3 × 1 g/day of Ceftazidim (cephalosporin). 

On the third day, the patient complained of angina-type pain accompanied by severe dyspnea. The cardiologist rises the suspicion of an ST elevation anteroseptal myocardial infarction (STEMI)/myocarditis requesting a transfer to a superior county hospital. During the transfer, she presented with a GCS of 15 points, respiratory frequency of 12/min, heart rate of 62 bpm, blood pressure of 113/78, O_2_ saturation of 99%, and angina and minor abdominal pain. She was admitted with the following diagnostics: “Acute myocardial infarction STEMI KILLIP 1, Chronic ischemic heart disease, Hypertension gr. I, Pregnancy 34–35 weeks, SARS COV2 infection” accusing angina. 

The admission EKG stated normal sinus rhythm, a heart rate of 60 bpm, an intermediate QRS axis, and an elevated ST segment in the anterior territory associated with under-leveling of the inferior one. Laboratory analysis found hs-cTnI at 12,000 ng/mL. Echocardiography found undilated left and right ventricles, left ventricular systolic function slightly depressed, EFLV 45%, akinesia of the apex of the septum and left ventricle, second-degree mitral regurgitation, and no free pericardial space. The anterior-descending branch of the left coronary artery was found to have an acute dissection with a 25 mm subocclusive stenosis in II and III segments and TIMI II distal flow. The circumflex coronary artery and the right coronary are both free of angiographic abnormalities. Under the shield of double antiaggregants and efficient anticoagulant use, direct stenting is performed using a DES 3 × 33 mm stent with the re-establishment of the coronary lumen covering the initial part of the dissection, with a good outcome, TIMI III flow being achieved distally. During the interventional coronarography, the patient was hemodynamically stable without inotropic medication, but at the end of the procedure, she complained of abdominal pain, for which an emergency gynecological consult was requested. At this moment, she was without angina, without left ventricular insufficiency, hemodynamically and electrically stable, with a blood pressure of 108/80 mmHg, a heart rate of 60 bpm, and an O_2_ level of 95% without a mask. After 5 h without any symptoms, she suddenly presented with a cardiac arrest with pulseless ventricular tachycardia that responded after 1 min of CPR with brief cardiac activity (atrial fibrillation) and after another minute with a second cardiac arrest. At this point, she is intubated and ventilated; she is administered Adrenaline and after 5 min of CPR, she presents ventricular fibrillation, for which she receives 2 shocks of 200 J each and begins to receive Adrenaline, and 300 mg of Cordarone every 4 min. After 30 min of CPR without a positive outcome, an emergency c-section is decided to save the fetus. She is rushed under CPR maneuvers to the gynecology operating room, where a c-section is performed still under CPR maneuvers without response. Death is declared after 6 h from the initial stenting. 

**Figure 1:**
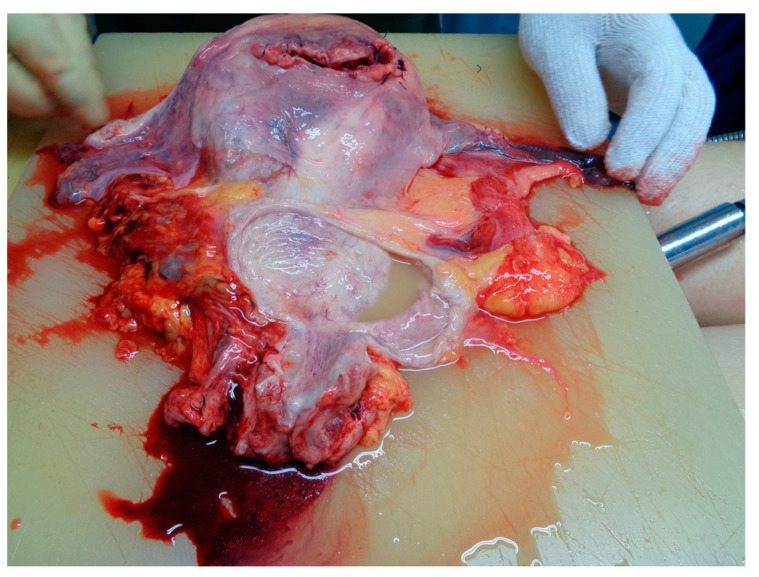
Autopsy aspect of the uterus and annexes.

According to Romanian Law, a medico-legal autopsy was requested by the inquirer (SCD under 24 h of hospitalization and medico-surgical treatment). The autopsy revealed a Caucasian female of 167 cm in height and 56 kg in weight. There was no evidence of congenital abnormalities, disease conditions, or trauma, signs of treatment or surgery (from iv treatment, resuscitation, or c-section). All organs were placed in normal anatomical positions; the frontal part of the fundus of the uterus (Figure 1) presented a horizontal incision of 10 cm sutured with 8 stitches, and the uterus had 17 × 15 × 5 cm with a thickness of 1.5 cm. 

**Figure 2:**
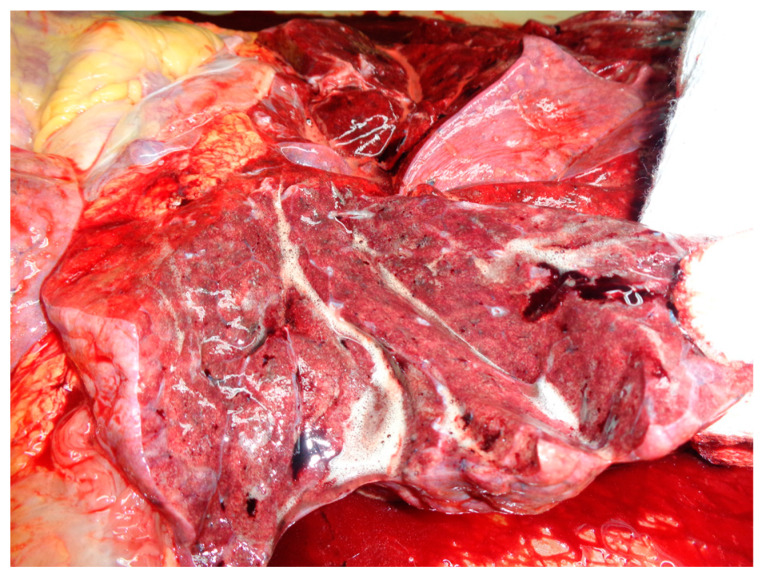
Autopsy aspect of acute pulmonary edema.

The thoracic cavity presented 200 mL of citrine serum liquid in both pleural cavities, with sternal fractures due to mechanical resuscitation. After sectioning the lungs, there were macroscopic signs of acute pulmonary edema (Figure 2). 

**Figure 3:**
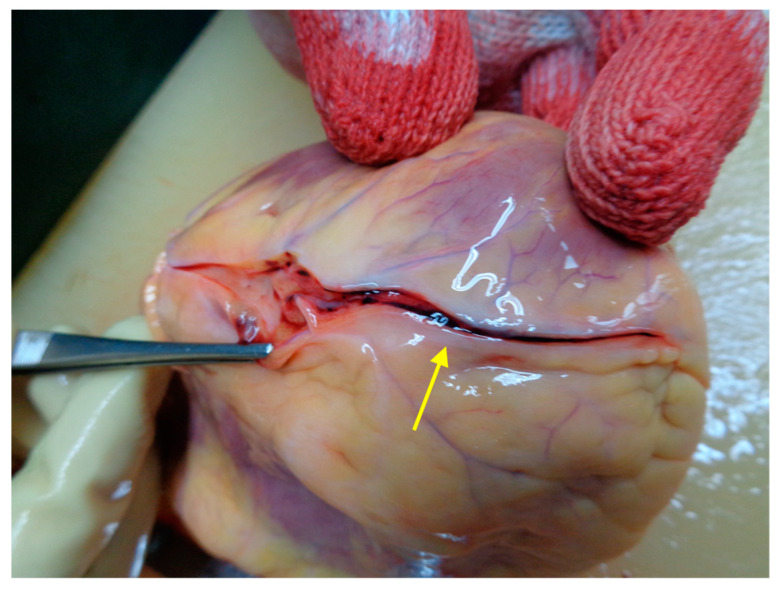
Autopsy aspect of the anterior-descending artery (acute wall dissection with stenosis in segments II and III).

**Figure 4:**
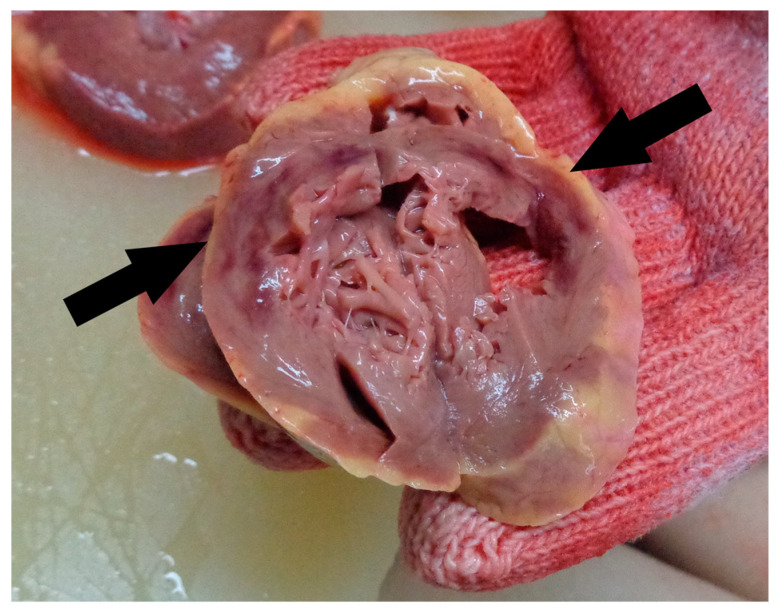
Autopsy aspect of the ventricles (acute myocardial infarction is marked with an arrow).

The heart weighed 320 g, measured 13 × 13 × 5.5 cm in dimension, and had a normal valvular aspect. The epicardial adipose tissue was 0.5–0.7 cm in thickness (for the right and left coronaries). Moreover, at the level of the anterior-descending artery, we identified an acute wall dissection with stenosis in segments II and III, as shown in Figure 3. The dimensions of the ventricular walls were 1.1 × 1.2 × 0.2 cm (left ventricle × septum × right ventricle), and the cavities were not dilated. On myocardial slides, we observed myocardosclerosis as thin white-pearly plots in the muscle tissue, which can be observed in Figure 4, with a pale subendocardial aspect circumferentially, in the anteroseptal and posterior walls of the left ventricle, with 2 hemorrhagic areas of 4 × 1 × 5 cm and 3.5 × 1.5 × 5.5 cm.

**Figure 5:**
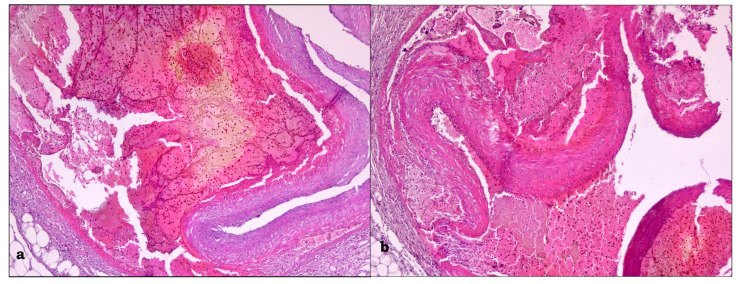
(**a**) Large IMH (left) compressing the true lumen (right); (**b**) the visible intimal tear surrounded by an inflammatory cellular infiltrate.

The circumflex artery had a slight hypoplasia (0.3–0.4 cm compared with 0.6 cm of the other coronaries) with no visible atherosclerosis; the anterior-descending artery had a stent of 3 cm with an unobstructed lumen; after removing it, a discontinuity of the wall was observed with hemorrhagic infiltrate surrounding it. Microscopically, as seen in Figure 5, the transmural acute myocardial infarction was confirmed. The anterior-descending artery examination revealed a discontinuous intimal area and a massive haemorrhage into the subjacent media. No pathological changes in the artery wall were seen.

It was concluded that the death was non-violent (pathological) due to acute pulmonary edema as a consequence of an acute myocardial infarction from an acute coronary dissection in a SARS-COV2-infected person.

### 2.2. Case 2

A 38-year-old female in apparently good health condition, pregnant at 38/39 weeks, with normal fetus development (one previous pregnancy without problems), with no pathological background, no history of smoking or alcohol consumption, and who checked all her mandatory antenatal visits as normal, suddenly complained of intense chest pain. Five months before the angina attack, she and her child had COVID-19 in asymptomatic forms. She was brought to a county hospital with severe retrosternal chest pain of, and because of the maternal-fetal endangerment, after the cardiological and gynecological consultation, she was transferred to a different county hospital with the diagnosis: “II pregnancy, 38/39 weeks. Viable fetus in the right occipital-iliac cranial presentation. Intact fetal membranes. Eutocic pelvis. The onset of labor. Fibrocicatricial uterus after C-section. Acute anterior myocardial infarction. Chronic ischemic heart disease. Cimented prosthesis of the right hip joint”. 

Immediately after admission, she underwent an emergency c-section, with a newborn male baby weighing 3930 g with an Apgar score of 7 at 1 min and 9 at 5 min. After the c-section, she was rushed to the interventional cardiology department, where the coronarography performed found acute coronary dissections of the anterior-descending artery with occlusive stenosis and acute dissection of the circumflex branch, for which two stents were mounted and transferred to the intensive care unit of the gynecology department. After this procedure, she was intubated, sedated, and hemodynamically stable, with a well-contracted uterus, under continuous perfusion with Oxistin 10 IU, and diuresis was present (400 mL). 

She was extubated the next morning, conscious and cooperative after extubation, with a blood pressure of 88/50 mmHg, a heart rate of 115 bpm, and a 100% O_2_ level with O_2_ addition through a mask. The patient’s condition deteriorated abruptly one hour after extubating, with tachypnea, bilateral rhonchi, and pearl-pink sputum as symptoms of severe pulmonary edema. Promptly, she underwent cardiac reassessment and initiated therapeutic intervention comprising Furosemide, Morphine, and non-invasive ventilation (she had an 85% O_2_ level). Nitroglycerin was not administered due to her hypotension (80/40 mmHg). She did not react to non-invasive ventilation with fast desaturation, so she was intubated and mechanically ventilated, given therapy with Dopamine, and then Dobutamine and Noradrenaline was started. The cardiac ultrasound exam highlights severely depressed left ventricle function with 20–25% ejection fraction, global hypokinesia, akinesia at the apex of the left ventricle, and 3rd-degree mitral valve insufficiency. Without a positive response from the treatment, she presented a clinical pattern of refractory cardiogenic shock, remaining hemodynamically unstable, blood pressure of 65/45 mmHg, a heart rate of 160 bpm, and O_2_ levels at 65% intubated/mechanically ventilated with 100% O_2_ added. She underwent another cardiac ultrasound examination, which did not show any improvement from the previous one. A total of 10 h after the stenting, she presented with pulseless electrical activity, and after one hour of CPR with a total of 10 mg of Adrenaline being administered, she was declared dead.

A medico-legal autopsy was requested by the inquirer (SCD under 24 h of hospitalization and medico-surgical treatment) according to the same law.

**Figure 6:**
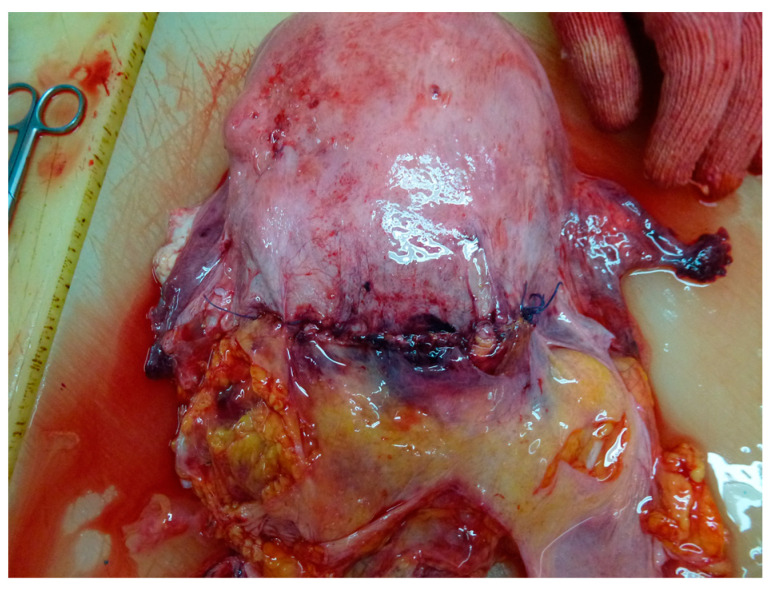
Autopsy aspect of the uterus.

The autopsy revealed a Caucasian female of 170 cm tall and weighing 72 kg. There was no indication of congenital anomalies, but there was one 20 cm vertical scar with the sign of 19 stitches on the lateral aspect of the left hip, as well as signs of treatment and surgery (from iv treatment, resuscitation, and c-section). All organs were placed in their proper anatomical position; the frontal-upper part of the fundus of the uterus presented a horizontal incision of 13 cm that was constantly sutured (Figure 6), and the uterus measured 18 × 16 × 5.5 cm with a thickness of 1.5 cm. 

**Figure 7:**
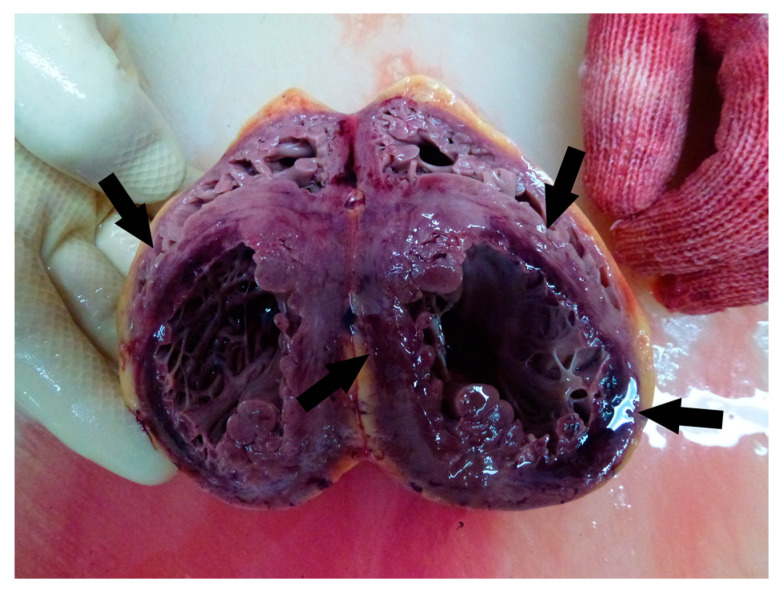
Autopsy aspect of the ventricles (acute MI is marked with an arrows).

The thoracic cavity had sternal and rib fractures due to mechanical resuscitation. The lungs were heavy, and after sectioning, there were macroscopic signs of acute pulmonary edema and acute stasis. A total of 50 mL of yellowish liquid was found in the pericardial sac. The heart weighed 450 g and measured 12 × 12 × 5.5 cm in dimension, after sectioning, with normal valvular aspect and globally dilated ventricles. The epicardial adipose tissue was 0.6–0.8 cm in thickness (for the right and left coronaries). The dimensions of the ventricular walls were 0.8 × 0.8 × 0.3 cm (left ventricle × septum × right ventricle). On myocardial slides, we observed pale, ischemic tissue, myocardosclerosis as thin white-pearly plots, and in the anterolateral wall of the left ventricle, a diffuse hemorrhagic area of 8 × 1 cm that was visible from the base to the apex (Figure 7).

**Figure 8:**
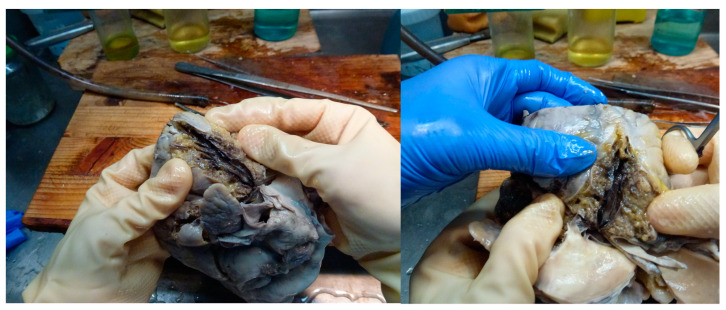
Autopsy aspect of the anterior-descending coronary (acute wall dissection with stenosis in segments II and III).

**Figure 9:**
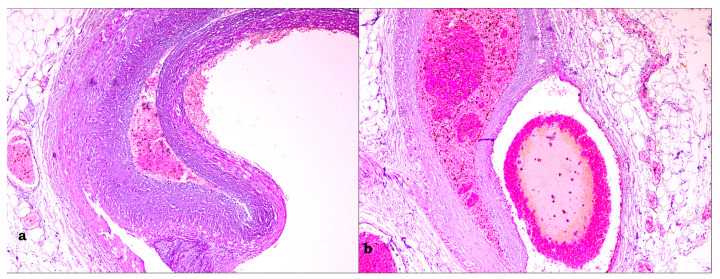
(**a**) The area of dissection (right) and the compressed lumen (left)—IMH was removed during macroscopic orientation; (**b**) the dissection also involved the small arterial branches.

The heart was placed in 10% formaldehyde for one week before being opened to analyze the coronaries. The circumflex artery had hypoplasia from the emergence (0.3 cm compared with 0.6 cm of the other coronaries) and at 3.2 cm suddenly dilated at 0.7 cm with the presence of a thrombus of 2 cm in length with no visible atherosclerosis and a visible thinning of the wall. The anterior-descending artery (Figure 8) had, right after emergence, a stent of 2.5 cm with a thrombosed obstructed lumen, followed by another stent with an identical image. After removing them, a discontinuity of the wall was observed with hemorrhagic infiltration in the tunica media of the coronary. Microscopically, the acute myocardial infarction was confirmed. On all the serial sections examined from the level of the circumflex and anterior-descending arteries (Figure 9), an intramural hematoma (IMH) was revealed occupying the dissection area of the tunica media and compressing the true lumen. The smaller branches of the arteries were involved in dissection. No intimal tear was observed. The presence of an inflammatory cellular infiltrate surrounding the dissection was very helpful in excluding an iatrogenic postmortem dissection. Rare, and small, fibrous, and lipid-rich atherosclerotic plaques were identified.

It was concluded that the death was non-violent (pathological) due to acute pulmonary edema as a consequence of an acute myocardial infarction from an extensive acute coronary dissection.

## 3. Discussion

SCAD represents one of the rare causes of myocardial infarction and SCD. Like SCD, the etiology, prognosis, and possible treatment of this pathology are not well established, making it a rare but important cause of acute myocardial infarction in young, active patients, especially postpartum women. The true prevalence of SCAD is uncertain because the pathology is often misdiagnosed. 

Regarding the etiology of SCAD, in the past, DeMaio et al. [13] underlined three sub-groups: the first group with undiagnosed coronary atherosclerosis, the second one in the postpartum period, and the third without a predisposing factor. If in the first group, SCAD was considered the result of a junction disruption between the intima and media due to a plaque rupture, and in the second group, the etiology of the dissection during the postpartum state was unknown, with microstructural changes due to hemodynamic and hormonal factors being implied [14]. 

Future research should better define this condition, excluding iatrogenic, traumatic, and atherosclerotic dissection, and describe it as a coronary obstruction caused by the formation of an IMH or an intimal disruption. Recent studies have suggested that SCAD may be the cause of acute coronary syndrome in up to 35% of cases of MI in women aged <50 years and is the most common cause of pregnancy-associated MI [6,15]. This statement is based on the advances made in intravascular imaging techniques [16] and the development of SCAD-specific angiographic classification [17].

Regarding the coronary distribution of SCAD, any artery can be affected, but the left anterior descending artery is the most commonly affected (32–46% of cases) [18,19,20,21,22]. SCAD with multiple coronary vessel obstructions was mentioned in 9–23% of the studied cases [18,19,20,21,22,23]. The spontaneous formation of IMH within the wall of a coronary artery has been confirmed by intracoronary imaging [24,25,26,27] and histopathological findings [28,29,30,31,32].

In the development of SCAD, two theories are still in debate, both being borrowed from the aneurysm etiopathogenesis: the first, which states that a disruption in the vessel wall (intimal tear) will allow the blood from the true lumen to enter and generate a false lumen [33], and the second, which mentions, as a primary cause, a spontaneous hemorrhagic event arising from the vasa vasorum within the vessel wall [34].

Several studies [31,32,35,36] have described a periadventitial inflammatory infiltrate, with eosinophils being predominant; some consider this condition to be pathognomonic [35,37,38], while others see it as a nonspecific response to a vascular injury [28]. Fibromuscular dysplasia (FMD) described in histopathological reports involving the coronaries in which SCAD occurred suggested that coronary FMD was the cause of SCAD [39,40,41,42,43,44]. FMD is, by definition, a nonatherosclerotic, noninflammatory vascular disease that can affect nearly any arterial wall and can manifest as stenosis, aneurysm, or dissection.

Most pregnancy-related SCAD events occur in the third trimester or early postpartum period, with the left anterior-descending artery being the most commonly affected. It is believed that estrogen and progesterone receptors found in the coronary arteries may weaken the vessel wall, which culminates in coronary wall rupture, IMH, and the onset of clinical symptoms [15]. Advanced maternal age and age at first childbirth, chronic hypertension, dyslipidemia, gestational hypertension, and preeclampsia were reported to increase the risk of SCAD [9,18]. The average age of pregnancy-associated SCAD is between 33 and 36 years. In the study conducted by the Mayo Clinic [45], 54 women with pregnancy SCAD were compared with 269 women with non-pregnancy SCAD. The pregnancy SCAD cases were younger, more likely to present an ST-segment–elevation MI (50% versus 36%; *p* = 0.013), had left main and multivessel dissections, had lower left ventricle function, and were less likely to have a concurrent FMD.

In addition to pregnancy hormonal changes, other potential hormone-mediated SCAD triggers are still under discussion (perimenopausal state, oral contraceptives, postmenopausal hormone therapy, infertility treatments) [46,47,48]. Briller et al. mentioned prothrombotic changes happening in pregnancy (complement activation, the release of proinflammatory cytokines, antigen-antibody abnormal responses, prothrombotic phenomena, or endothelial-vascular dysregulations) also found in the immune-mediated severe forms of COVID-19 [49]. Mercedes et al. [50], in a case series of 154 pregnant women, admitted with COVID-19 infection checked cardiac biomarkers, with Tn elevations in 9.7%. Median Tn levels were 34.6 ng/mL (IQR: 14.4–55.5 ng/mL). Most had ventricular dysfunction and elevated pro-B-type natriuretic peptide concentrations (209 pg/mL [IQR: 184–246 pg/mL]). The mortality rate was 13.3%. In their study, Long et al. showed that in nonpregnant women, COVID-19 is associated with several cardiovascular complications, including myocardial injury and myocarditis, AMI, heart failure, and dysrhythmias. Some of the medications utilized to treat COVID-19 also have potential cardiac complications [51]. We found only a few case reports in which patients had advanced maternal age, no or few CV risk factors, were COVID-19 positive, and needed emergency C-sections due to an acute coronary syndrome, with no mention of the outcome of the patients [52,53]. Interestingly, the patients were in the 32–36-year age frame, and were previously healthy individuals with no or minimal CV risk factors, making a firm statement about the impact of COVID-19 in the CV system. It is worth mentioning that two studies showed that the prevalence of acute myocardial injury in patients with COVID-19 increased patients’ mortality significantly more than age or previous CV risk factors [54,55]. 

The traditional angiographic description of SCAD noted the presence of multiple radiolucent lumens and extraluminal contrast staining (intraluminal filling defects) [56]. In his research, Saw [17] proposed and implemented an angiographic SCAD 3 type classification: type 1—the classic appearance of multiple radiolucent lumens or arterial wall contrast staining; type 2—the presence of diffuse stenosis that can be of varying severity and length, with 2A (diffuse arterial narrowing bordered by normal segments proximal and distal to the IMH) and 2B (diffuse narrowing that extends to the distal tip of the artery); type 3—focal or tubular stenosis, usually <20 mm in length, which mimics atherosclerosis, and, in this case, intracoronary imaging is required to confirm the presence of the IMH.

Aquaro et al. performed post-mortem cardiac magnetic resonance imaging (PM-CMR) using formalin-fixed explanted hearts from 115 cases of sudden death in their study. When using a guideline-driven histological sample, the PMCMR interpretation matched the final forensic diagnosis in 93 cases (81%) (88% sensitivity; AUC of 0.77). With the addition of a PMCMR-driven strategy, the matching went up to 102 cases (89%) (sensitivity of 89%; AUC of 0.88). The authors found that PMCMR exhibited a high degree of accuracy in identifying the cardiac etiology of sudden death and might be used as a legitimate adjunct for forensic diagnosis [57]. In addition, in another study by Baronti et al., performed on five individuals with SCD who had previously received mRNA vaccinations, the PM-CMR revealed ischemic lesions and coronary artery stenosis in all cases [58].

The American College of Cardiology/American Heart Association and the European Society of Cardiology guidelines for the management of ACS agreed on an early invasive strategy with immediate revascularization of the diagnosed lesions over conservative therapy alone [59,60]. This stent-based lesion protocol reduced the risk of a recurrent occlusion at the lesion site in atherosclerotic MI, but regarding ACS caused by SCAD, future studies are needed. Pathological coronaries in SCAD tend to have weak lumens, which can increase the risk of an iatrogenic dissection PCI.

Coronary guidewires may enter the false lumen and occlude the true lumen. Balloon dilation and stent placement can also increase the risk of extending the intimal dissection in addition to changing the IMH position upstream or, even worse, downstream, producing a distal vessel obstruction that can lead very quickly to SCD.

An accurate diagnosis of SCAD will ensure that an invasive strategy of percutaneous coronary intervention (PCI) is reserved only for a properly selected group, given that PCI for SCAD is associated with lower technical success and higher complications than PCI for atherosclerotic disease [61,62,63].

## 4. Conclusions

Despite the latest advances in this topic, SCAD continues to be misdiagnosed, underdiagnosed, and managed as an atherosclerotic acute coronary syndrome, which may harm patients with SCAD. Thus, by the traditional angiographic description of SCAD (multiple lumens or contrast staining of arterial walls), >70% of SCAD cases will, unfortunately, be missed. We consider that getting accustomed to the diffuse, narrowed-lumen appearance of IMH and the use of intracoronary imaging may improve the future diagnosis of SCAD.

## Data Availability

Not applicable.
